# Reduced Excitability and Increased Neurite Complexity of Cortical Interneurons in a Familial Mouse Model of Amyotrophic Lateral Sclerosis

**DOI:** 10.3389/fncel.2018.00328

**Published:** 2018-09-28

**Authors:** Rosemary M. Clark, Mariana Brizuela, Catherine A. Blizzard, Tracey C. Dickson

**Affiliations:** ^1^Menzies Institute for Medical Research, University of Tasmania, Hobart, TAS, Australia; ^2^Flinders Medical Centre, Flinders University, Adelaide, SA, Australia

**Keywords:** excitability, structure, interneuron, cortex, SOD1 G93A mutant

## Abstract

Cortical interneurons play a crucial role in regulating inhibitory-excitatory balance in brain circuits, filtering synaptic information and dictating the activity of pyramidal cells through the release of GABA. In the fatal motor neuron (MN) disease, amyotrophic lateral sclerosis (ALS), an imbalance between excitation and inhibition is an early event in the motor cortex, preceding the development of overt clinical symptoms. Patients with both sporadic and familial forms of the disease exhibit reduced cortical inhibition, including patients with mutations in the copper/zinc superoxide-dismutase-1 (SOD1) gene. In this study, we investigated the influence of the familial disease-causing hSOD1-G93A ALS mutation on cortical interneurons in neuronal networks. We performed whole-cell patch-clamp recordings and neurobiotin tracing from GFP positive interneurons in primary cortical cultures derived from Gad67-GFP::hSOD1^G93A^ mouse embryos. Targeted recordings revealed no overt differences in the passive properties of Gad67-GFP::hSOD1^G93A^ interneurons, however the peak outward current was significantly diminished and cells were less excitable compared to Gad67-GFP::WT controls. *Post hoc* neurite reconstruction identified a significantly increased morphological complexity of the Gad67-GFP::hSOD1^G93A^ interneuron neurite arbor compared to Gad67-GFP::WT controls. Our results from the SOD1 model suggest that cortical interneurons have electrophysiological and morphological alterations that could contribute to attenuated inhibitory function in the disease. Determining if these phenomena are driven by the network or represent intrinsic alteration of the interneuron may help explain the emergence of inhibitory susceptibility and ultimately disrupted excitability, in ALS.

## Introduction

Amyotrophic lateral sclerosis (ALS) is the most common and severe form of motor neuron (MN) disease. It is clinically characterized by selective loss of the upper and lower MNs in the primary motor cortex and spinal cord, resulting in progressive motor system failure and death within 3–5 years of diagnosis (Talbot, 2014; Brown and Al-Chalabi, 2017). This currently incurable disease is clinically heterogeneous and its etiology remains unknown. However, accumulating evidence from several clinical and experimental studies suggests the disease pathogenesis may center on altered regulation of MN excitability (Turner and Kiernan, 2012; Clark et al., 2015; Geevasinga et al., 2016).

In both sporadic and familial forms of ALS, patients have been found to present with neurophysiological alterations described as hyperexcitability (Vucic and Kiernan, 2006; Vucic et al., 2008; Geevasinga et al., 2015). Originating in the motor cortex and preceding detectable lower MN dysfunction and symptom onset (Menon et al., 2015), it is proposed that hyperexcitability enhances the susceptibility of MNs to cell death through glutamatergic excitotoxicity (Blizzard et al., 2015; Eisen et al., 2017). In ALS, hyperexcitability likely results from dysfunctional inhibition exerted by GABAergic interneurons, as well as intrinsic changes to sodium (Na^+^) and potassium (K^+^) channel function on MNs (Geevasinga et al., 2016; Do-Ha et al., 2018).

Evidence for ion channel dysfunction is highlighted by a convergent hyperexcitability phenotype in patient-derived MNs, including *TARDBP*, *C9ORF72* and superoxide-dismutase-1 (*SOD1*) mutation carriers (Wainger et al., 2014; Devlin et al., 2015). TDP-43 and SOD1 ALS rodent models identify changes in the excitability of MNs prior to symptoms (Fogarty et al., 2015; Handley et al., 2017). However, more recent studies recognize progressive alterations in the number and excitability of interneurons throughout the disease course in TDP-43 and SOD1 models (Zhang et al., 2016; Clark et al., 2017; Kim et al., 2017). Clinical imaging studies indicate loss of inhibitory activity is a common and early feature of cortical hyperexcitability (Menon et al., 2015), and a key determinant of clinical disease progression (Shibuya et al., 2016). As such, there is a growing body of evidence to suggest dysregulated inhibition, presumably mediated by cortical interneurons, may drive an excitatory/inhibitory imbalance in ALS.

Here, we focused on the potential for the familial hSOD1^G93A^ mutation to influence firing properties and morphology of cortical interneurons in Gad67-GFP::hSOD1^G93A^ cultures. Structural alteration of pyramidal neurons is demonstrated in hSOD1^G93A^ studies (Jara et al., 2012; Fogarty et al., 2016; Saba et al., 2016), but few studies report changes to the morphological fine structure of interneurons (Clark et al., 2017). Additionally, mutant SOD1 is theorized to mediate non-cell autonomous pathogenicity through perturbed function of multiple cell types early in development (Kuo et al., 2004; van Zundert et al., 2008; Martin et al., 2013; Wainger et al., 2014; Devlin et al., 2015). As such, we hypothesize that the hSOD1^G93A^ mutation can perturb the excitability and neurite structure of cortical interneurons in culture.

## Methods

### Animals

All procedures were approved by the Animal Ethics Committee of the University of Tasmania (#A0013586) and conducted in accordance with the Australian Code of Practice for the Care and Use of Animals for Scientific Purposes. Gad67-GFP knock-in transgenic mice (Tamamaki et al., 2003) express green fluorescent protein (GFP) under the interneuron-specific *Gad67* promoter. High copy number hSOD1^G93A^ mice were maintained and fully backcrossed to the C57BL/6 background (Gurney et al., 1994; B6.Cg-Tg(SOD1^G93A^)1Gur.J^1^, Jackson Laboratories, Bar Harbor, ME, USA).

### Cortical Culture Electrophysiology

Primary cortical cultures were prepared by carefully dissecting individual mouse embryo neocortices to enrich for neurons as previously described (Brizuela et al., 2015, 2017), with some modifications. Cells were plated at 3.5 × 10^4^ cells/mm^2^ for up to 12 days and genotyped for GFP (Brizuela et al., 2015) and the hSOD1^G93A^ mutation (Leitner et al., 2009). Whole-cell voltage and current clamp recordings were performed as previously described (Brizuela et al., 2015), with minor modifications. Glass capillaries (impedance 7–9 Ω) were filled with intracellular solution containing neurobiotin for *post hoc* cell identification. Voltage responses to current injection were recorded from the cell’s resting potential (applying 25 pA steps for 200 ms from −100 pA to 500 pA). Recordings were filtered at 5 kHz and sampled at 10 kHz and terminated when access resistance was ≥15 MΩ. Voltage-gated sodium and potassium currents were investigated as previously described (Brizuela et al., 2015). Data was analyzed using the programs Igor (WaveMetrics, USA) and Axograph (Axograph Scientific, Australia).

### Immunocytochemistry

Cultures were processed for immunocytochemistry as previously described (Brizuela et al., 2015). Primary antibodies (rat anti-GFP, 1:3,000, Nacalai tesque, RRID: AB_10013361). Secondary antibodies (Alexa Fluor anti-rat 488, 1:1,000, Molecular Probes, RRID: AB_2534074). Neurobiotin-filled interneurons were labeled with streptavidin-546 and imaged using a UltraView Spinning disc confocal microscope (Perkin Elmer) with Velocity Software (Velocity v6 3.0, 2013, Perkin Elmer).

### Morphological Analyses

Cell processes were traced through Z-stack series (5 μm, 0.5 μm intervals) with Neurolucida™ (MBF Bioscience, VT, USA) and assessed using branched structure and sholl analyses with Neurolucida Explorer 11 (MBF Bioscience). Interneurons that met electrophysiology inclusion criteria were morphometrically assessed.

### Statistical Analysis

All statistical analysis was performed in GraphPad Prism (Version 6.0c, GraphPad Software La Jolla, CA, USA). Unless otherwise stated, comparisons utilized Mann-Whitney tests, after applying d’Agostino and Pearson’s normality test, results expressed as median with interquartile range. Sholl analysis and current-frequency relationships used two-way ANOVA. Data was considered significant at *p < 0.05.

## Results

### Membrane Properties and Reduced Excitability in Cultured Gad67-GFP::hSOD1^G93A^ Interneurons

Previous studies have demonstrated that mutant hSOD1^G93A^ can perturb neuronal excitability during development, which may contribute to cellular vulnerability in disease (Kuo et al., 2004; van Zundert et al., 2008; Martin et al., 2013; Wainger et al., 2014; Devlin et al., 2015). Here, we focus on investigating cortical interneuron excitability in neuronal culture by examining firing patterns and membrane properties of interneurons in Gad67-GFP::hSOD1^G93A^ cultures.

**Figure 1:**
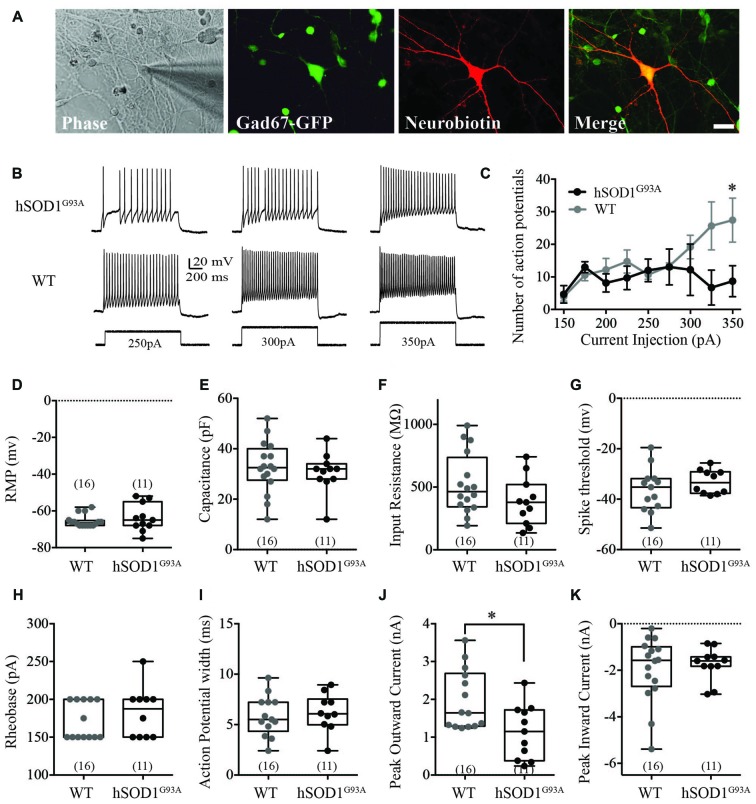
Electrophysiological characterization of Gad67-GFP positive hSOD1^G93A^ cortical interneurons *in vitro*. Primary neuronal cultures were prepared from E15.5 Gad67-GFP::hSOD1^G93A^ embryos as described in “*Methods*” section. **(A)** Whole cell patch-clamped interneurons were positive for Gad67-GFP and *post hoc* labeled for neurobiotin-streptavidin-546. **(B,C)** Representative voltage traces **(B)** and current-spike frequency relationship **(C)** measured from GFP-positive interneurons in 12 DIV cortical culture (Gad67-GFP::hSOD1^G93A^, *n* = 11 cells from five cultures; Gad67-GFP::WT, *n* = 16 cells from five cultures; *p < 0.05, two-way ANOVA), error bars show mean ± SEM. **(D–K)** The active and passive electrophysiological properties of GFP-positive interneurons, including: the resting membrane potential (RMP; **D**), capacitance **(E)**, input resistance **(F)**, threshold to fire **(G)**, rheobase **(H)**, action potential (AP) width **(I)**, peak inward current **(J)** and significantly decreased peak outward current (**K**; Gad67-GFP::hSOD1^G93A^, *n* = 11 cells from five cultures; Gad67-GFP::WT, *n* = 16 cells from five cultures; *p < 0.05, Mann-Whitney test). Box-and-whisker plots show the interquartile range.

We used whole-cell recordings from multipolar cortical interneurons to characterize the effect of the hSOD1^G93A^ mutation on cortical interneuron excitability in the presence of synaptic currents. Selection of GFP-positive neurons ensured studied cells were interneurons, with neurobiotin labeling used for *post hoc* identification (Figure 1A). We found the hSOD1^G93A^ mutation significantly affected firing properties of cortical interneurons, decreasing the number of action potentials (APs; Figure 1B) in response to 200 ms depolarizing current step injections at 350pA (Figure 1C; 8.675 ± 2.752pA for hSOD1^G93A^, 27.42 ± 3.888pA for WT; *p* < 0.05, two-way ANOVA, Bonferroni *post hoc*; *F*_(8,47)_ = 2.305, *p* = 0.0357, interaction between genotype and frequency in two-way ANOVA). There was no significant difference in passive electrophysiological properties, including the resting membrane potential (RMP; Figure 1D; −66.5 mv, −67.30 to −63.82 for WT (*n* = 16) v −63.73, −68.75 to −58.71 for hSOD1^G93A^ (*n* = 11), *p* > 0.05), capacitance (Figure 1E; 32.50 pF, 27.17–38.20 for WT (*n* = 16) v 32.00, 25.39–35.89 for hSOD1^G93A^ (*n* = 11), *p* > 0.05) and input resistance (Figure 1F; 463 MΩ, 394.1–652.1 for WT (*n* = 16) v 378.0, 255.9–513.4 for hSOD1^G93A^ (*n* = 11), *p* > 0.05). Further alterations in excitability were not detected through investigation of AP characteristics, including the spike threshold (Figure 1G; −35.24 mv, −41.59 to −31.07 for WT (*n* = 16) v −33.43, −36.52 to −29.64 for hSOD1^G93A^ (*n* = 11), *p* > 0.05), the minimal stimulus required to initiate an AP (Figure 1H; rheobase, 150.0 pA, 156.2–186.1 for WT (*n* = 16) v 187.5, 158.6–206.4 for hSOD1^G93A^ (*n* = 11), *p* > 0.05) and the AP duration (Figure 1I; 5.504 ms, 4.649–7.086 for WT (*n* = 16) v 6.066, 4.849–7.563 for hSOD1^G93A^ (*n* = 11), *p* > 0.05). However, investigation of voltage-dependent currents identified a significant decrease in the peak outward current (Figure 1J; 1.644 nA, 1.524–2.442 for WT (*n* = 16) v 1.151, 0.6636–1.624 for hSOD1^G93A^ (*n* = 11), *p* < 0.05), while there was no significant difference in the peak inward current (Figure 1K; −1.575 nA, −2.691 to −1.207 for WT (*n* = 16) v −1.598, −2.214 to −1.273 for hSOD1^G93A^ (*n* = 11), *p* > 0.05). Taken together, this data suggests that cortical interneurons are less excitable and have decreased peak outward currents in the presence of the hSOD1^G93A^ mutation.

### The Morphology of Cortical Interneurons Is Changed in Gad67-GFP::hSOD1^G93A^ Cultures

GABAergic interneurons have dynamic axonal structures that can alter in response to changes in activity, to modify post-synaptic targets (Flores and Méndez, 2014). Given the role of interneuron morphology in shaping inhibition during development (Huang, 2009; Le Magueresse and Monyer, 2013), we determined whether there was evidence of changes to the interneuron neurite arbor in Gad67-GFP::hSOD1^G93A^ cultures.

**Figure 2:**
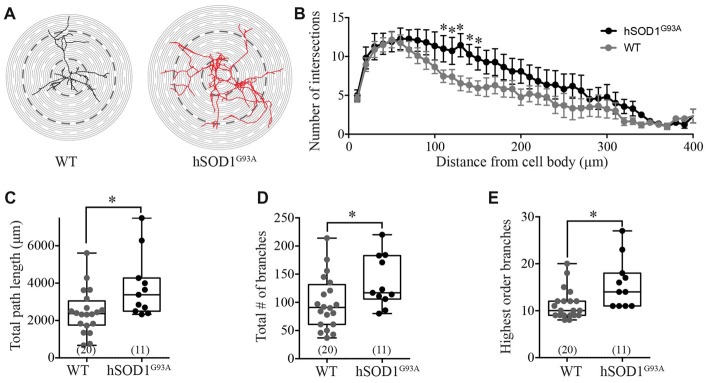
Morphological characterization of Gad67-GFP positive hSOD1^G93A^ cortical interneurons *in vitro*. **(A)** Representative images of patched interneurons reconstructed from *post hoc* neurobiotin-streptavidin labeling in Gad67-GFP::hSOD1^G93A^ and Gad67-GFP::WT cultures. Each concentric circle represents 10 μm, and each dashed line represents 50 μm from the cell soma. **(B)** Sholl analysis denoting the morphological complexity of GFP-positive interneurons as measured by the average number of neurites intersecting with concentric circles placed at 10 μm intervals from the cell soma (Gad67-GFP::hSOD1^G93A^, *n* = 11 cells from five cultures; Gad67-GFP::WT, *n* = 20 cells from five cultures; *p < 0.05, two-way ANOVA), error bars show mean ± SEM. **(C–E)** Histograms quantifying significantly increased total neurite path length (μm; **C**), total branch number **(D)** and average number of branches **(E)** of Gad67-GFP::hSOD1^G93A^ interneurons compared to Gad67-GFP::WT controls (Gad67-GFP::hSOD1^G93A^, *n* = 11 cells from 5 cultures; Gad67-GFP::WT, *n* = 20 cells from five cultures; *p < 0.05, Mann-Whitney test). Box-and-whisker plots show the interquartile range.

Interneuron morphology was characterized by tracing the three-dimensional structure of neurobiotin-filled GFP-positive interneuron processes in WT and hSOD1^G93A^ cultures. Initial examination revealed a distinct increase in the complexity of hSOD1^G93A^ interneurons (Figure 2A). Assessing the characteristics of the interneuron neurite field with sholl analysis (intersections per concentric shell placed at 10 μm radiating outward from the soma), we identified a significant increase in the number of processes on hSOD1^G93A^ interneurons at 110–150 μm from the cell soma compared to WT (*p* < 0.05, two-way ANOVA, Bonferroni *post hoc*; *F*_(1,849)_ = 24.77, *p* = 0.0001, main effect of genotype in two-way ANOVA; see Figure 2B). The distance from the soma also independently influenced the neurite arbor complexity (*F*_(40,849)_ = 10.58, *p* = 0.0001, main effect of distance in two-way ANOVA). To investigate the increased complexity of hSOD1^G93A^ interneurons we next used branched structural analyses. As shown in Figure 2C, the path length was significantly increased in hSOD1^G93A^ interneurons (2363 μm, 1914–3020 for WT (*n* = 20) v 3380, 2673–4929 for hSOD1^G93A^ (*n* = 11), *p* < 0.05). However, there was also a significant increase in total branch numbers (Figure 2D; 91.00, 76.65–121.0 for WT (*n* = 20) v 117.0, 105.1–166.7 for hSOD1^G93A^ (*n* = 11), *p* < 0.05) and the highest order of branches on hSOD1^G93A^ interneurons (Figure 2E; 10.00, 9.77–12.83 for WT (*n* = 20) v 14.0, 12.16–19.30 for hSOD1^G93A^ (*n* = 11), *p* < 0.05). These results suggest that the presence of the hSOD1^G93A^ mutation can result in early inhibitory arbor structural remodeling in cortical networks.

## Discussion

This study is the first investigation of changes in the excitability and neurite arborization of cortical interneurons in neocortical embryonic cultures from a SOD1 rodent model. The principal finding is that excitability of cultured cortical interneurons may be altered by the hSOD1^G93A^ mutation, and that changes to the complexity of the neurite structure accompany this effect *in vitro*. Specifically, attenuation of excitability is supported by differences in the current-voltage relationship between WT and hSOD1^G93A^ mouse interneurons, and by the observation of reduced peak outward currents in hSOD1^G93A^ interneurons. Interestingly, outward potassium currents are affected in MNs derived from SOD1 ALS patients (Wainger et al., 2014), and by mutant SOD1 oligomers (Zhang et al., 2017), which could account for the changes in the hSOD1^G93A^ cortical interneurons.

However, a number of studies highlight increased excitability of cortical pyramidal neuron populations both *in vivo* and *in vitro* (Pieri et al., 2009; Fogarty et al., 2015), which could drive changes in the morphological development of cortical interneurons. The inhibitory neurite arbor can undergo activity-dependent structural remodeling (Bartolini et al., 2013; Babij and De Marco Garcia, 2016) and the level of network activity can produce subtle but significant changes to interneuron morphology (Schuemann et al., 2013). Importantly, in networks deprived of activity, cortical interneurons have been shown to extend collaterals beyond their normal projection range (Marik et al., 2010). While the data presented here are correlational not causal, altered structural and electrophysiological properties of cortical interneurons provide evidence that hSOD1^G93A^ could disturb the inhibitory/excitatory balance in developing neuronal networks, early in the disease pathogenesis.

Previous studies highlight progressive and differential interneuron involvement in the hSOD1^G93A^ mouse model (Clark et al., 2017; Kim et al., 2017). In particular, Kim et al support altered interneuron excitability in hSOD1^G93A^ models, finding progressive hyperexcitability with disease progression *in vivo* (Kim et al., 2017). In the current study we find evidence for intrinsically hypoexcitability. These data support a role for the hSOD1^G93A^ mutation in the early perturbation of inhibitory neuron populations in the disease. However, to determine the effect on functional inhibition, future studies should establish if synaptic excitability is changed to compensate for abnormal firing properties which could normalize firing rates (Turrigiano, 2008). In addition, further experimentation will be required to delineate if this is the cause or consequence of perturbed excitatory neuron excitability in the disease.

## Data Availability

All data generated or analyzed in this study are available from the corresponding author upon request.

## Author Contributions

RC performed cortical culturing, genotyping, immunocytochemistry, data analysis and was a major contributor in writing the manuscript. MB performed electrophysiology and data analysis. CB, MB and TD contributed to experimental design, drafting and editing of manuscript. All authors read and approved the final manuscript.

## Conflict of Interest Statement

The authors declare that the research was conducted in the absence of any commercial or financial relationships that could be construed as a potential conflict of interest. The reviewer SG and the handling Editor declared their shared affiliation.

## Footnotes

^1^http://www.jax.org/strain/004435
